# *Porphyromonas gingivalis* Placental Atopobiosis and Inflammatory Responses in Women With Adverse Pregnancy Outcomes

**DOI:** 10.3389/fmicb.2020.591626

**Published:** 2020-12-02

**Authors:** Luz Amparo Gómez, Juliette De Avila, Diana Marcela Castillo, Daniel Antonio Montenegro, Tammy Gorety Trujillo, Lina J. Suárez, Gloria Inés Lafaurie

**Affiliations:** ^1^ Unit of Basic Oral Investigations-UIBO, School of Dentistry, Universidad El Bosque, Bogotá, Colombia; ^2^ Cellular and Molecular Immunology Group, School of Dentistry, Universidad El Bosque, Bogotá, Colombia; ^3^ School of Medicine, Universidad El Bosque, Bogotá, Colombia; ^4^ Basic Science and Oral Medicine Department, School of Dentistry, Universidad Nacional de Colombia, Bogotá, Colombia

**Keywords:** pregnancy outcome, cytokines, periodontitis, dysbiosis, *Porphyromonas gingivalis*, macrophage, inflammation, placenta

## Abstract

The microbiome modulates inflammation at the fetal maternal interface on both term and preterm labor. Inflammophilic oral bacteria, such as *Porphyromonas gingivalis*, as well as urogenital microorganisms (UGM) could translocate to the placenta and activate immune mechanisms in decidual tissue that is associated with adverse pregnancy outcomes (APO). This study establishes the associations between the presence of microbes in the placenta and placental cytokine patterns in women who presented APO, e.g., low birth weight (LBW), preterm premature rupture of membranes (PPROM), preterm birth (PTB) and other clinical signs related to Chorioamnionitis (CA). A total of 40 pregnant women were included in the study and divided into five groups according to placental infection (PI) and APO, as follows: (1) women without PI and without APO (*n* = 17), (2) women with *P. gingivalis*-related PI and APO (*n* = 5), (3) women with *P. gingivalis*-related PI and without APO (*n* = 4), (4) women with PI related to UGM and APO (*n* = 5) and (5) women without PI with APO (*n* = 9). Obstetric, clinical periodontal status evaluation, and subgingival plaque sampling were performed at the time of delivery. Placental levels of interleukin IL-1β, IL-6, IL-10, IL-15, IL-17A, IL-17F, IL-21, IL-12p70, tumor necrosis factor-α (TNF-α), monocyte chemoattractant protein-1 α (MCP-1α), granzyme B, and interferon-γ (IFN-γ) were determined using a multiplex flow cytometry assay. All patients showed a predominant Th-1 cytokine profile related to labor, characterized by IFN-γ overexpression. The analysis by groups suggests that Th-1 profile was trending to maintain cytotoxic cell activity by the expression of IL-15 and granzyme B, except for the group with *P. gingivalis*-related PI and APO, which exhibited a reduction of IL-10 and IL-17F cytokines (*p* < 0.05) and a Th-1 profile favoring macrophage activation by MCP-1 production (*p* < 0.05). This study confirms a pro-inflammatory pattern associated with labor, characterized by a Th-1 profile and the activity of cytotoxic cells, which is enhanced by PI with UGM. However, PI associated with *P. gingivalis* suggests a switch where the Th-1 profile favors an inflammatory response mediated by MCP-1 and macrophage activity as a mechanistic explanation of its possible relationship with adverse outcomes in pregnancy.

## Introduction

Given the concept that the uterus is a sterile microenvironment, the description of the presence of a healthy microbiome, typical of the placenta, has been widely debated since 2014 by different studies, both in favor (Aagaard et al., 2014; Doyle et al., 2014; Parnell et al., 2017; Seferovic et al., 2019) and against the hypothesis (Leiby et al., 2018; Theis et al., 2019). In fact, those who mentioned the presence of a placental microbiome, characterized its composition as being very similar to the human oral microbiome, i.e., composed of non-pathogenic commensals, such as Firmicutes, Tenericutes, Proteobacteria, Bacteroidetes, and Fusobacteria phyla (Aagaard et al., 2014). However, the role of the microbiome in host response have been explained by the release of metabolic products in adverse pregnancy outcomes (APO), like preterm birth (PTB), low birth weight (LBW), and preterm premature rupture of membranes (PPROM; Parris et al., 2020). It has even been determined that bacterial DNA and short-chain fatty acids produced by commensals in utero have the potential to influence the development of the fetal immune system (Stinson et al., 2019), just as uterine quiescence during pregnancy is mediated by anti-inflammatory mechanisms (Mendelson, 2009).

Nowadays, there is a lack of consensus regarding the definition of the terms chorioamnionitis (CA) or intra-amniotic infection, and a of specific clinical signs and symptoms of intra-amniotic infection even if diagnosed with the gold standard of culture/PCR in amniotic fluid (Morgan, 2016). Nevertheless, statistical analyses of APO associated with intra-amniotic infection by certain microorganisms, have established that intra-amniotic infection may be, not only one of the main risk factors for PTB, but also a direct cause by the force, temporality and the gradient with which the DNA of microorganisms is associated with PTB (DiGiulio et al., 2008).

The abundance of certain species with clinical relevance differs between placentas after spontaneous PTB, non-spontaneous PTB, and term birth and supports the fact that a significant percentage of placentas prone to spontaneous PTB has a component of intrauterine infection (Leon et al., 2018). The frequencies of microorganisms in the intrauterine microenvironment are highly variable, which could be related to the type of samples taken, their processing and the techniques used to detect them or their DNA; these frequencies are also related to the gestation stage at which the APO is presented. The numbers can reach up to 60% in the early stages and fluctuate between 10–25% in the third quarter (DiGiulio et al., 2008; Combs et al., 2014; Morgan, 2016). Specifically, in the placenta, microorganisms can invade the amnion and chorion as well as the villous tree, which is frequently associated with APO including congenital infections (Theis et al., 2019).

The intrauterine microbiome can come from different sites and get there by different routes: it may arrive (1) ascending from the vagina through small channels in the cervical mucus; (2) by the hematogenous route; (3) by transmembrane filtration from the intestine to the peritoneal cavity with retrograde ascension *via* the fallopian tubes; (4) from the intestine or from the blood; or (5) from leukocytes and dendritic cells carrying bacteria and spreading them to other locations such as the uterus (Bardos et al., 2020). The hematogenous route can also be used by microorganisms of urinary and respiratory infections, and from the oral cavity (Cao et al., 2014; Prince et al., 2016).

Periodontitis is an inflammatory non-communicable disease that develops in susceptible subjects from the actions of dysbiotic microbial communities and pathobionts that exhibit synergistic virulence that potentiates the host response, while promoting their own survival by cross feeding from the products of inflammation related to tissue destruction. Periodontitis may eventually lead to tooth loss and systemic complications (Hajishengallis, 2014), including pregnancy complications (Hajishengallis, 2015).

*P. gingivalis* is considered a keystone pathogen in the pathogenesis of periodontitis. This type of pathogens supports and stabilizes microbiota associated with disease states and has the capacity to cause inflammation even if present in insignificant quantities (Hajishengallis et al., 2012). These bacteria are the most frequent microorganisms present during bacteremia in patients with periodontitis (Lafaurie et al., 2007; Horliana et al., 2014) and they are the most common microorganisms in the amniotic fluid and placental tissue (León et al., 2007; Hasegawa-Nakamura et al., 2011; Vanterpool et al., 2016). This translocation of microorganisms to blood or other tissues has also been described as dysbiosis, so the term atopobiosis has been coined to describe the translocation of microorganisms in order to avoid confusion. Thus, atopobiosis describes the appearance of bacteria at sites other than its usual location and is associated with multiple chronic, non-communicable, and inflammatory diseases (Potgieter et al., 2015).

Whether commensals participate or not in the onset of birth, there is always an inflammatory response that leads to delivery, which includes innate and acquired immune responses; in fact, inflammatory responses are part of pregnancy itself. During pregnancy estrogen and progesterone favor the humoral immune response; estrogens stimulate the production of antibodies and the activation of natural killer (NK) cells and macrophages and they decrease the production of proinflammatory cytokines such as interleukin (IL)-1β and tumor necrosis factor-α (TNF-α). Progesterone may inhibit lymphocyte activation and reduce Th-1 profile cytokines [IL-1β, IL-2, IL-12, IL-15, IL-18, interferon-γ (IFN)-γ, and TNF-α], so an increase in the levels of these hormones may be involved in maternal-fetal nonrejection (Piccinni et al., 2000). However, at the end of the third trimester, a pattern of Th-1-type cytokines predominates since an inflammatory environment is necessary for the onset of labor (Saito et al., 2010).

The presence of several cytokines in the amniotic fluid has been reported as an inflammatory marker to predict the risk of preterm delivery (Liu et al., 2017a). Th-1-type cytokines (IFN-γ, IL-12p70, IL-15, and IL-18) can be over-regulated by infection and proinflammatory cytokines such as TNF-α, IL-1β, IL-6, and IL-21 may participate in the adverse outcomes of pregnancy (Erlebacher, 2013). Another less studied mechanism is related to the abnormal regulation of NK cells by natural cytotoxicity receptors, which regulate NK cell cytotoxicity and cytokine production or the activation of decidual macrophages by MCP-1 and other profiles of cytokines such as the Th-17 profile, which have been implicated in APO such as PTB and PPROM (Fukui et al., 2009; Bardou et al., 2014; Liu et al., 2017b).

A complex interaction between infection and inflammation on both the systemic and intrauterine environments and various biological processes between pathogen and host seem to be crucially involved in the pregnancy outcomes. However, the mechanisms of the immune activation pathways and the triggers of the immune response at the molecular level, associated with induction of preterm labor are not clear. Causal infectious agents, the role of polymicrobial infections, critical sites of infection, as well as the participation of cells and activation pathways of the immune response have barely been identified (Cappelletti et al., 2016).

Chopra et al. (2020) based on the evidence, suggest the atopobiosis of *P. gingivalis* to the amniotic fluid and placenta of women with APO; however, the molecular mechanisms by which *P. gingivalis* relates to the occurrence of complications during pregnancy and in the newborn are not clear in the literature (Chopra et al., 2020). Some of the potential pathogenic mechanisms which might link *P. gingivalis* and APO include: (1) Direct invasion, translocation, and injury to the foetal-placental unit/interface and maternal tissues, (2) Persistence and survival within the foetal and maternal tissues and immune response evasion, (3) Increased production of proinflammatory cytokines and shift in maternal-foetal immune response from Th2 to Th1 with the onset of Th17/T regulatory cell imbalance, (4) Activation of the acute-phase response, (5) Onset of Polymicrobial dysbiosis and development of pathobiont species, (6) Increased oxidative stress in the foetal and maternal tissue, and (7) Increased foetal adrenal cortisone production and the onset of foetal stress (Chopra et al., 2020).

Based on the occurrence of APO and the presence or absence of placental infection (PI), this study seeks to establish a possible relationship between the presence of PI by *P. gingivalis* and urogenital microorganisms (UGM) and the pattern of cytokine expression in the placenta (IFN-γ, IL-12p70, IL-17A, IL-17F, IL-23, IL-21, IL-10, IL-15, granzyme B, MCP-1, TNF-α, IL-1β, and IL-6) which may be related to the occurrence of APOs (e.g., LBW, PPROM, and PTB) and other clinical signs related to CA.

## Materials and Methods

This study involved a group of subjects from a larger previously published case control study (Montenegro et al., 2019) whose objective was to stablish the association between oral and UGM in the placenta and PTB, PPROM, and clinical signs of intraamniotic infection. All participants agreed to participate in the study and signed an informed consent approved by the Institutional Ethics Committee of Universidad El Bosque, PCI-2014-18.

### Study Population

A total of 224 pregnant women aged ≥18 years that were admitted for delivery at the Service of Gynecology and Obstetrics of Hospital Simon Bolivar in Bogotá, Colombia from August 2014 to August 2016 were initially enrolled in the case control study of oral and urogenital intra-amniotic infection in women with preterm delivery (Montenegro et al., 2019), in which women included should have been monitored in the hospital during their pregnancy and have a detailed record of prenatal care, especially records for the presence or absence of associated infection, vaginosis, and CA. The diagnosis of clinical CA was based on the Gibbs and Duff antepartum criteria and on the clinical criteria of the abnormal appearance of post-delivery membranes postpartum. All of the patients that received attention at the emergency department of Simon Bolivar Hospital provided their relevant medical history, where symptoms of a potential PTB or intra-amniotic infection were identified (i.e., uterine activity, uterine hypersensitivity, expulsion of mucus plug and/or poor genital bleeding, maternal or fetal tachycardia, and fever). Women with antibiotic intake 1 month before delivery, uterine abnormalities, fetal malformations, twin pregnancy, heavy vaginal bleeding, fetal distress, infectious or systemic diseases such as HIV, tuberculosis, candidiasis or diabetes, smokers or users of alcohol or psychoactive substances, and women with premature termination of pregnancy for medical reasons were excluded. If the placental sample was contaminated in the operating room during delivery or the sample could not be analyzed due to its quantity, the woman was not included; six women were excluded for these reasons.

For cytokine analysis, a second phase was carried out and the patients were selected based on the results of first phase specially the presence of infection and perinatal condition. In order to control the influence of age and other reasons of inflammation, all women older than 40 years and patients with pre-eclampsia were excluded.

**Figure 1:**
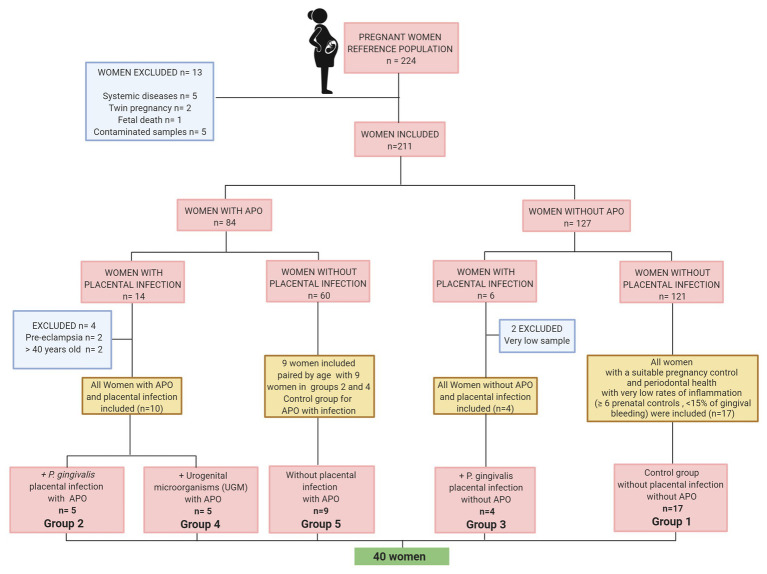
Flow chart of sample selection.

Figure 1 shows a flowchart describing the reference population, selection and exclusion of participants to establish the cytokine analysis groups according to APO and placental UGM/*P. gingivalis* infection. The groups were distributed as follows:

Group 1 – Control group for APO and PI. All women without APO, with a suitable pregnancy control (≥ 6 prenatal controls) and periodontal health with very low rates of inflammation evidence (<15% of gingival bleeding) *n* = 17.
Group 2 – *P. gingivalis*-related PI with APO. All women with PI associated with *P. gingivalis* with APO (one aged >40 years was excluded) *n* = 5.
Group 3 – *P. gingivalis*-related PI without APO. All women with PI associated with *P. gingivalis* without APO (two samples could not be analyzed due to its quantity) *n* = 4.
Group 4 – UGM-related PI with APO. All women with PI associated with UGM with APO (three participants were excluded due to preeclampsia or >40 years old) *n* = 5.
Group 5 – No PI with APO. nine women with APO without PI paired by age with nine women with APO/PI including infection by *P. gingivalis* and by UGM only. *n* = 9 (control group for APO with placental infection).

### Adverse Pregnancy Outcome Assessment

PTB was established for those births that occurred before 37 weeks of gestation, and LBW was established for infants weighing <2,500 g. PPROM was established for those patients who had >12 h from rupture to delivery. Clinical signs of PI were identified as fever, maternal tachycardia, fetal tachycardia, and fetid fluid (Gibbs and Duff, 1991).

### Periodontal Clinical Examination and Subgingival Plaque Sampling

Two calibrated periodontists (properly trained before the beginning of the study), who were blinded regarding the group category at the time of the evaluation, performed the periodontal assessment of all patients before or up to 8 h after delivery. A full-mouth examination was performed at six sites on each permanent tooth using a North Carolina® probe (PCPUNC-15; Hu-Friedy, Chicago, IL, United States). The indices employed for the diagnosis were: probing depth (PD), clinical attachment level (CAL), bleeding on probing (BOP), plaque index, and gingival index (GI). The presence of periodontitis was determined according to the criteria established by the Centers for Disease Control and Prevention/American Academy of Periodontology (≥ 2 interproximal sites with CAL ≥ 3 mm and ≥ 2 interproximal sites with PD ≥ 4 mm; not on the same tooth; Eke et al., 2010).

The supragingival plaque was removed, and absorbent paper points were inserted for 20 s into the six deepest periodontal sites of each subject for polymerase chain reaction (PCR) detection of *P. gingivalis*, *Tannerella forsythia*, *Treponema denticola*, *Eubacterium nodatum*, *Aggregatibacter actinomycetemcomitans*, and *Fusobacterium nucleatum* in the subgingival plaque.

### Placental Tissues Sample Collection

After delivery, two samples of amniotic membrane (1.5 cm^2^) were taken: one at the umbilical cord insertion base and the second at the free edge of the contralateral cotyledon of the first sample, which were transferred to an empty sterile microcentrifuge tube and refrigerated at −20°C until processing. One sample was used for extraction and amplification of bacterial DNA in the placenta and the other one for tissue cytokine profile detection in patients with PI.

### Extraction and Amplification of Bacterial DNA in the Placenta

The disintegration of the placenta samples was performed until total tissue trituration was achieved; each sample was weighed in triplicate under sterile conditions and deposited in a 1.5 ml microcentrifuge tube and 80 μl phosphate-buffered saline (PBS) was added to homogenize in vortex. All tubes with the samples were submerged in liquid nitrogen for 5 min to achieve a complete lysis of the tissue. After thawing, 100 μl buffer ATL, 3 μl proteinase K, and 16 μl lysozyme were added and mixed in vortex until complete homogenization was achieved. The samples were incubated with shaking at a temperature of 56°C for 24 h until complete lysis was achieved and vortexed again.

To extract DNA, the QIAamp DNA Mini Kit (Qiagen, Hilden, Germany) was used. Before DNA amplification, the purity and amount of DNA were obtained using NanoDrop 2000 (Thermo Scientific, Rockford, IL, United States). Bacterial DNA amplification was performed by nested PCR using a universal primer for Eubacteria for the first amplification EUB27F: 5'-GAG TTT GAT CCT GGC TCA G-3' and EUB1544R: 5'-AGA AAG GAG GTG ATC CAG CC-3', directed to the amplification of a fragment of the 16S rRNA gene, common among the bacteria. The amplification was evidenced by the presence of the amplification product (1407 bp) in agarose gel (2%) with 0.5 μg/ml ethidium bromide. The amplification of the specified DNA for each microorganism was done using the PCR product obtained from the amplification as the template for the universal primer of oral bacteria such as *P. gingivalis*, *T. forsythia*, *T. denticola*, *A. actinomycetemcomitans*, *Campylobacter rectus*, and *Prevotella intermedia* (Ashimoto et al., 1996), *Parvimonas micra*, and *F. nucleatum* (Kuriyama et al., 2000), and the microorganisms associated with urogenital infection such as *Gardnerella vaginalis* (Fredricks et al., 2007), *Ureaplasma urealyticum* (Teng et al., 1994), *Mycoplasma hominis* (Blanchard et al., 1993), and *Candida albicans* (Mahmoundi-Rad et al., 2012). Those included in the DNA of reference strains of the American Type Culture Collection (Manassas, VA, United States) were used as positive controls and sterile water was used as negative control. The identification of each microorganism was confirmed by the presence of amplification products using agarose gel electrophoresis (1.5%) and 0.5 μg/ml ethidium bromide staining.

### Cytokine Profile Analysis

#### Dissection of Placenta and Tissue Cytokine Profiles Detection

The placental samples were homogenized by mechanical disaggregation of tissues using the Medimachine System (120V; BD™, San Jose, CA, United States). The samples were cut in small pieces, which were inserted into a Medicon of 35 μm (BD™ Medimachine Medicon, Sterile) by adding 10 μl protease-free PBS (Sigma, St. Louis, MO, United States) for 3 cycles at 80 rpm. The homogenized samples were passed through Filcons of 20 μm (BD™ Medimachine Filcon, Sterile, Cup-Type), a washing cycle at 1800 rpm for 5 min was done, and the supernatants were recovered to measure the soluble cytokine levels using a multiplex bead-based LEGENDplex™ assay by flow cytometry. A Human Th Cytokine mix and match 13 plex Panel (San Diego, CA, United States) was customized, in which 13 capture beads of two different sizes were used, each one conjugated with antibodies against IFN-γ, IL-12p70, IL-17A, IL-17F, IL-23, IL-21, IL-10, IL-15, granzyme B, MCP-1, TNF-α, IL-1ß, or IL-6. The samples were processed according to manufacture instructions in a sandwich immunoassay. The flow cytometry acquisition was carried out in a BD FACS Accuri™ C6 Plus using the BD Accuri™ C6 Plus software; the analysis was performed based on a standard curve into a logistic regression model (five points; BioLegend’s LEGENDplex™ Software, 2016). The results were expressed in pg/ml units.

### Statistical Analysis

In order to find correlations among cytokine profile groups and APO, a Factorial analysis of mixed data (FAMD) was performed that combined Principal Component Analysis (PCA) for continuous variables and Multiple Correspondence Analysis (MCA) for categorical variables (Audigier et al., 2016). Kruskal-Wallis/Mann-Whitney U test was used to compare the cytokines between the different groups. One-way ANOVA with Dunnett’s *post-hoc* test was performed to compare the relationship between cytokine/IFN-γ among groups. Spearman rank based pairwise correlation analysis was performed to analyze cytokine abundances in all the groups. R statistical software and IBM-SPSS version 23 were used for all analyses. Heatmaps were made using Morpheus, https://software.broadinstitute.org/morpheus.

## Results

### Sociodemographic Characteristics of the Study Population

In total 40 patients were included to assess the cytokine response and the association with atopobiosis of *P. gingivalis* to placental tissue (Figure 1). The sociodemographic analysis shows homogeneity among groups (Table 1). There were no significant differences in age, marital status, and schooling between groups (*p* > 0.05).

**Table 1:** Socio-demographic characteristics and periodontal condition according to the presence of placental infection (PI) and adverse pregnancy outcomes (APO).

		Control	*Pg* + APO+	*Pg* + APO-	UGM + APO+	PI‐ APO+
		*n* = 17	*n* = 5	*n* = 4	*n* = 5	*n* = 9
Age mean ± SD		21.29 ± 3.1	24.60 ± 4.6	19.75 ± 1.7	22.20 ± 3	19.33 ± 1.2
Civil status (F%)						
Single		5 (29.4)	0	2 (50)	1 (20)	2 (22.2)
Married		1 (5.9)	0	0	0	1 (11.1)
Civil union		11 (64.7)	5 (100)	2 (50)	4 (80)	6 (66.7)
Education (F%)						
Elementary		0	0	0	0	2 (22.2)
High school		15 (88.2)	4 (80)	4 (100)	5 (100)	6 (66.7)
Technical		2 (11.8)	1 (20.0)	0	0	1 (11.1)
Periodontitis n (%)						
Yes^**^		0 (0.0)	3 (60.0)	3 (75.0)	2 (40.0)	0 (0.0)
Clinical signs of placental infection (F%)						
Yes		0 (0.0)	2 (40.0)	0 (0.0)	0 (0.0)	0 (0.0)

** *p* < 0.05 statistically significant difference among groups by Chi square test. Clinical signs of infection included uterine hypersensitivity, fever, maternal tachycardia, fetal tachycardia, and fetid flow.

APO, adverse pregnancy outcome; UGM, urogenital microorganisms; SD, standard deviation; IQR, Interquartile range; *Pg*+ APO+, *P. gingivalis* (+) adverse pregnancy outcome (+); *Pg*+ APO–, *P. gingivalis* (+) adverse pregnancy outcome –; UGM+ APO+, urogenital microorganisms (+) adverse pregnancy outcome +; PI– APO+, Placental infection (−) adverse pregnancy outcome (+); Control, adverse pregnancy outcome (−) without placental infection.

### Periodontal Clinical Characteristics and Identification of Microorganisms in Subgingival Plaque

Oral clinical assessment showed periodontitis presence in group 2 (*P. gingivalis*-related PI with APO), group 3 (*P. gingivalis*-related PI without APO) and group 4 (UGM-related PI with APO; Table 1), all of them associated with *P. gingivalis* presence in subgingival plaque (60, 75, and 40% respectively; Supplementary Table 1). In Patients with subgingival presence of *P. gingivalis* higher indices were more frequent and the highest median was observed in the *P. gingivalis*-related PI without APO (group 3) which presented a higher frequency of periodontitis (*p* < 0.05). No significant differences were observed for plaque index, PD, or CAL among groups (*p* > 0.05). Distribution of microorganisms in subgingival plaque is presented in Supplementary Table 1.

### Factorial Analysis of Mixed Data for Cytokine and Adverse Outcomes

**Figure 2:**
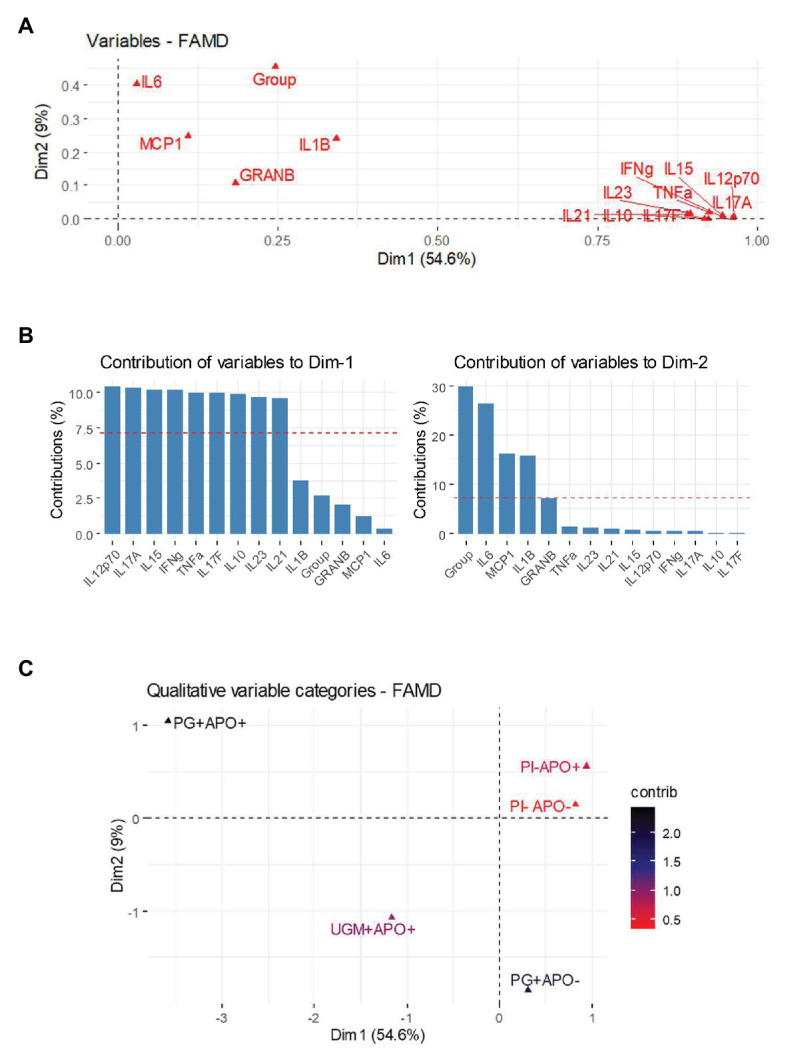
Factorial analysis of mixed data (FAMD) for cytokine and adverse outcomes. FAMD combines Principal Component Analysis (PCA) for continuous variables and Multiple Correspondence Analysis (MCA) for categorical variables in at least two dimensions. In this case Dim-1 represents those deliveries without APO and Dim-2 represents deliveries associated with APO. (A) shows a variables vector map as a geometric representation of variables presented by arrows where the length expresses the SD of each variable, and the angles among variables show their correlations (acute angles represent stronger correlations, right angles no correlations and straight/obtuse angles show inverse correlations). Note how cytokines from Th-1 [polarized to natural killer (NK) cell activation], Th-17 and T-reg are grouped in a network of acute angles showing a common behavior on Dim-1. However, cytokines associated with macrophage activation are leaning next to Dim-2 and near group variable. (B) shows a bar chart with the contribution of each cytokine to each dimension; the percentage could be understood as the weight of this variable in the dimension. In this way Dim-1 is dominated by Th-1 (polarized to NK cell activation) and Th-17 cytokines, while Dim-2 is polarized toward macrophage activation cytokines and group variable. The graphical representation of the components and the heat chart in (C) represent the final step in FAMD, showing an extraction to distinguish which group determines the principal contribution to each dimension. The results show a remarkable contribution of *P. gingivalis* placental infection and APO group to Dim-2.

Figure 2 shows the correlation between both quantitative and qualitative variables, and their contributions in two dimensions: 1 (without APO) and 2 (with APO). The contribution of the variables for delivery without APO show a pattern of cytokine expression predominantly inclined toward higher correlation among IFN-γ and NK related cytokines and secondary cytokines of Th17 profile, in contrast to the adverse outcomes dimension in which the correlations reveal that inflammatory cytokines associated with macrophage activation such as MCP-1, IL-6, and IL-1β are strongly correlated with the group variables (Figures 2A,B). To establish which group contributed to this correlation in Dim-2 with MCP-1, IL-6, and IL-1β, the FAMD allowed us to make an extraction by categories together with their contributions and correlations, showing that macrophage cytokines were mainly related to the *P. gingivalis*-related PI with APO (group 2), which was the group with the greatest contribution to the model representing a differential pattern associated to an activation of inflammatory response (Figure 2C).

### Analysis of Cytokine Profiles Between the Groups With or Without PI

**Figure 3:**
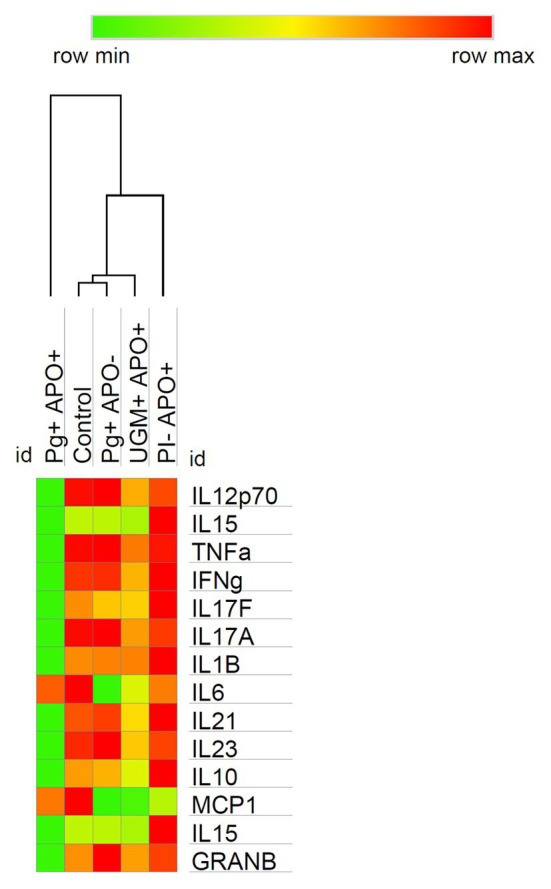
Cytokine profiles among groups analysis. The heat map shows the soluble cytokine concentrations in the controls and their variations among groups at time of delivery. Observe how the behavior in the profiling expression is similar in all groups except in the *P. gingivalis* PI with APO group which reflects a switch in two ways: (1) A significative decrease in the levels of cytokines associated with Th-1 (polarized to NK cell activation), Th-17 and T-reg, and (2) A maintenance of macrophage cytokine levels in contrast with the control group. It should be noted that in all cases the cytokine with higher levels of expression was IFN-γ which suggests the predominance of Th1 profile. Comparisons among groups were made by Kruskal-Wallis and Mann-Whitney U test with a confidence level of 95%. https://software.broadinstitute.org/morpheus.

**Figure 4:**
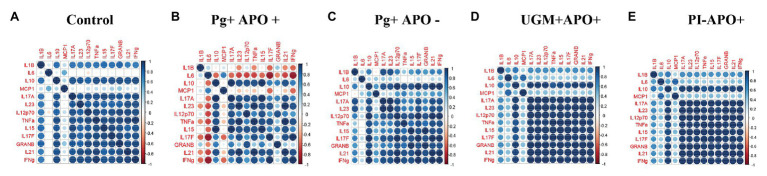
Correlation analysis between cytokines. Spearman rank based pairwise correlation analysis among cytokines (IL-1β, IL-6, IL-10, IL-15, IL-17A, IL-17F, IL-21, IL-12p70, TNF-α, MCP-1, granzyme-B, and IFN-γ) in **(A)** control, **(B)**, Pg + APO + **(C)** Pg + APO ‐ **(D)** UGM + APO+ **(E)** PI-APO+. The size of the spheres represents *p*-value. While strong correlations are shown by large circles, weak correlations are shown by small circles. The color of the circle denotes the strength of the correlation. Perfect positive correlation (with correlation coefficient 1) is indicated in dark blue, whereas perfect inverse correlation (with correlation coefficient 1) are colored in dark red.

Based on the previous results in which all profiles showed a similar behavior among the groups, except for the *P. gingivalis*-related PI with APO group, cytokine levels were analyzed according to Th-1, Treg and Th-17 profiles and compared by groups. As Figure 3 shows, this analysis allowed us to observe a generalized significant decrease in levels of the Th-1 (IFN-γ, IL-12p70, IL-15, Granzyme-B, TNF-α, and IL-1ß), Th-17 (IL-17F, IL-17A, IL-21, and IL-23), and Treg (IL-10) profile cytokines in the *P. gingivalis-*related PI with APO group when compared with the controls (*p* < 0.05). Meanwhile, macrophage activation cytokines (MCP-1 and IL6) in Th-1 profiling were maintained in both groups (*P. gingivalis-*related PI with APO and the controls), suggesting an important role for these cells in APO related to *P. gingivalis* placental infection. UGM infections did not show significant differences with the control group for any cytokine profile (Figure 3). Figure 4 shows an analysis of correlations between host cytokine levels in control, *P. gingivalis-related* PI with APO (Pg + APO+), *P. gingivalis*-related PI without APO (Pg-APO+), UGM-related PI with APO (UGM + APO+), and No PI with APO (PI-APO+) groups by Spearman’s rank-based correlations (Figures 4A–E). All groups showed high positive correlations (>0.6) between IFN-γ with IL-1β, IL-15, IL-17F, IL-21, IL-12p70, TNF-α IL-10, granzyme-B, and IL-17A but low correlations with MCP-1 and IL6. However, a high but negative correlation (> − 0.6) was observed in Pg + APO+ group between IFN-γ and MCP-1, IL-1β, and IL-6 levels. *P. gingivalis*-related PI with APO group showed also a negative correlation between IL-21, IL-17F with MCP-1, IL-1β, and IL6. High and negative correlations were found between all cytokines with IL6 in *P. gingivalis*-related infection with APO group but low correlations between all cytokines with MCP-1 and IL-1β.

### Cytokine/IFN-γ Ratio in Placental Tissue

**Figure 5:**
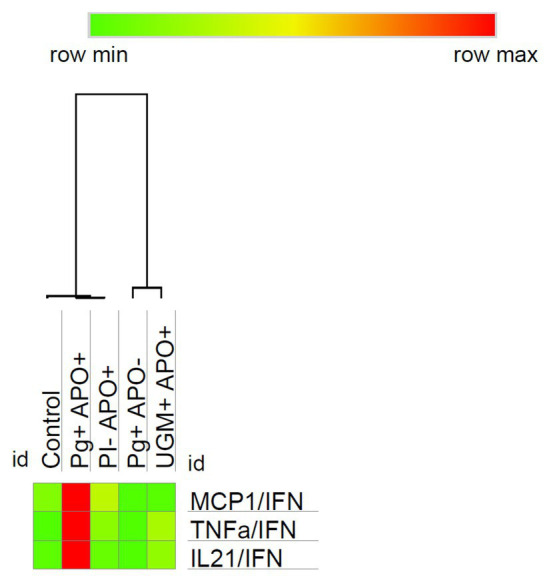
Relationship of MCP-1, TNF-α and IL-21 with IFN‐ γ. To identify the cell line polarized by Th-1 profile, the expression ratios for each cytokine were determined relative to the concentration of IFN-γ and were then compared between groups. As the heat map shows the *P. gingivalis* PI with APO group presents a particular profiling characterized by macrophage activation mediated *via* MCP-1/IFN-γ (*p* = 0.022), TNF-α/IFN-γ (*p* = 0.004), and IL-21/IFN-γ (*p* = 0.0001). Comparisons were done by one-way ANOVA with Dunnett’s post-hoc test with a confidence level of 95%. https://software.broadinstitute.org/morpheus.

Results showed that at the time of delivery the predominant profile was Th-1 in all of the groups. To identify the cell line in which that profile was polarized, the expression ratios for each cytokine were determined relative to the concentration of IFN-γ and were compared between groups, showing a common behavior of the activation of NK cells as the main target of Th-1 response in childbirth. *P. gingivalis*-related PI with APO group showed a variation in the response pattern, where MCP-1/IFN-γ, TNF-α/IFN-γ, and IL-21/IFN-γ showed differences with significantly higher concentrations (MCP-1/IFN-γ, *p* = 0.022; TNF-α/IFN-γ, *p* = 0.004; and IL-21/IFN-γ, *p* = 0.0001; Figure 5).

These results for *P. gingivalis*-related PI with APO group reflects high levels of IFN-γ with a significative decreasing of NK-related cytokines (*p* < 0.05) and the maintenance of those which are associated to macrophage activation when it was compared with the control group. In contrast, the other groups evaluated in this study presented a very similar pattern of cytokine expression characterized by high levels of IFN-γ and NK-related cytokines.

## Discussion

It is well known that during pregnancy there are multiple factors (hormonal, metabolic and immunological) that impact and influence the oral microflora; nevertheless, the role of oral atopobiosis in modulating inflammation in conditions like pregnancy is not yet clear since the oral microflora during gestation is still poorly characterized. This is even more relevant if the similarities of the placenta microbiome and that of the oral cavity are considered, which highlights the importance of atopobiosis of oral microorganisms in APOs (Balan et al., 2018).

The amniotic cavity of women with PTB has a much higher DNA content (more than previously believed) with a great diversity of microorganisms, including many that have not yet been characterized; The strength of these findings, due to the amount and timing in which they occur, could be useful in formulating a hypothesis to establish a causal association with APOs (DiGiulio et al., 2008); In the study from which our samples came from (Montenegro et al., 2019), the percentage of intrauterine infection was 9.47% (20/211), similar to that reported by previous studies, in which percentages varied between 10 and 25% in the third trimester of pregnancy (DiGiulio et al., 2008; Combs et al., 2014; Morgan, 2016).

*P. gingivalis* was the most prevalent identified oral microorganism in PI (12/211, 5.68%); however, in most cases it was not associated with the presence of clinical signs of intrauterine infection, although its frequency was higher in cases with APO (Montenegro et al., 2019). In the *P. gingivalis* PI cases, the presence of periodontitis linked to the subgingival presence of *P. gingivalis* was greater (60% for group 2 and 75% for group 3) and independent of the presence of APO. In group 4, where there was no PI due to *P. gingivalis*, the prevalence of periodontitis was lower and in the group with aseptic APOs, there was no periodontitis. Thus, not only the presence of the bacteria determines the occurrence of conditions or diseases linked to it. This would explain why not all the subjects with subgingival *P. gingivalis* have periodontitis or why not all the patients with PI with *P. gingivalis* show signs of clinical intrauterine infection or APO. Other factors, such as the amount of inoculum, virulence of the isolated microorganism, and individual immune response to the microorganism, could be associated with perinatal complications (Montenegro et al., 2019). *P. gingivalis* itself is not a proinflammatory pathogen, but by using different mechanisms it evades and alters components of the host’s immune response (disrupts innate immunity: TLRs and complement), which alters the development of the entire biofilm; then *P. gingivalis* exerts its role of “keystone pathogen” modulating the host response in susceptible individuals (Hajishengallis et al., 2012), which could explain why findings of *P. gingivalis* PI may not be always linked to the occurrence of APO (Supplementary Table 2).

Some studies suggest that intrauterine tissues in women with PTB or TB may have bacteria present without overt infection (Stout et al., 2013), but commensal bacteria can convert macrophages from the decidua to a proinflammatory state triggering labor. In other words, commensals present in the decidua trigger protease amplification of the inflammatory response by activating the resident macrophages (autocrine action) as well as other leukocytes, which would lead to signaling pathways being activated, resulting in the expression of genes from inflammation that starts labor. This raises the question of whether vaginal bacteria could be the initiators of term and preterm labor. Effaces of the cervix and its dilation can lead to the fetal membranes being exposed to the vaginal microbiome, which provides an entry route to the intrauterine microenvironment where they can initiate an inflammatory response that starts labor (Walsh et al., 2020).

The group with *P. gingivalis*-related PI with APO (group 2) showed clinical signs of intrauterine infection, which could indicate alterations in the mechanisms of inflammation in subjects susceptible to both infection and inflammation. There are multiple mechanisms described by which *P. gingivalis* could be related to the above. In the murine model, changes in gestational tissues have been observed after translocation of *P. gingivalis*, including areas of focused necrosis and increased inflammatory infiltrate. An increase in the expression of Fas, Fas-L and TLR2 at the placental level related to PTB is also observed in rats (Liang et al., 2018). On the other hand, the virulence of microorganisms in the placenta seems to depend on other factors, including genotype, bacterial load, host physiology, and environmental factors (Katz et al., 2009; Gomez-Arango et al., 2017; Fischer et al., 2019). Changes in the microbiome that are accompanied by significant variations in the microbial metabolic pathways as determined in integrative metagenomics analyses, can also contribute to the risk of PTB with or without severe CA (Prince et al., 2016).

Other bacterial species have also been identified in adverse pregnancy events. In this study, we identified PI by bacterial species of urogenital origin, such *M. hominis* and *C. albicans* in women with APO. *Mycoplasma hominis* and *Candida albicans* have been closely associated with APO such as PPROM, PTB, and LBW (Allen-Daniels et al., 2015; Farr et al., 2015). Infections by these microorganisms induced an immune response characterized by a significant increase in Th-1 proinflammatory profiles and NK cell activation. The inflammatory response mediated by IL-1β, TNF-α, PAF, IFN-γ, IL-6, PGE2, MMP-1, and MMP-9 causes the rupture of fetal membrane, increasing uterine contractions (Helmo et al., 2018). Meanwhile, women with *P. gingivalis* PI and APO showed a differential immune response characterized by macrophage activation that could explain the behavior of this infection in preterm delivery.

Although infection is a major risk factor for APO, it is possible that some outcomes occur in the absence of bacteria. In this study, in group 5 where the APO was aseptic, there was also no presence of periodontitis or presence in the subgingival plaque of the studied microorganisms. Actually, it has been reported that intra amniotic inflammation per se is associated with APO even in the absence of microorganisms; intrauterine colonization in the absence of inflammation appears to be relatively benign, but the presence of infection also seems to be related to the presence of inflammation, especially to increased levels of IL-6 that are related to increasing numbers of bacteria (Combs et al., 2014). On the other hand, most of these infections are subclinical and their diagnosis and treatment are difficult during the clinical course of the pregnancy (McCormick, 1985).

The analysis of the role of microorganisms in the host response at the uterine level in PTB, it has been stated that microorganisms in the uterus could increase the synthesis and release of proinflammatory cytokines, prostaglandins, and metalloproteinase, leading to cervical maturation, PPROM, uterine contractions, and finally PTB (Lannon et al., 2014). Normal pregnancy is marked by a state of increased inflammation throughout the gestation period that ends with the inflammatory cascade that begins labor. At conception, maternal immunity changes from a Th-1 dominated-proinflammatory state to a Th-2 immuno-tolerant response that allows fetal implantation and growth (Piccinni et al., 2000). A dysregulation of maternal immunity can lead to a placental invasion and a restriction of fetal growth leading to PTB, preeclampsia, and LBW. Although acute gestational stimuli such as infections are risk factors for PTB and LBW (Park et al., 2018), recent studies have shown that endogenous immune processes such as the presence of chronic inflammation can influence APO in the absence of infection (Ragsdale et al., 2019). At the end of the third trimester, a Th-1 pattern predominates, and some authors have considered that this change to an inflammatory environment is necessary to start delivery (Saito et al., 2010), as is corroborated by the results of this research. Thus, the present results support the cascade of events described in the literature for the occurrence of labor and we think that it can be precipitated by trigger events (e.g., infection, inflammation caused or not caused by microorganisms, stress, etc.); They are: (1) Activation of innate and adaptive immune cells with increased migratory activity, (2) Recruitment to the fetal maternal interface of activated cells mediated by the release of chemokines; and (3) Amplification of the inflammatory response by infiltrating leukocytes (Gomez‐ Lopez et al., 2014).

Additionally, in this study, IL-10 showed the lowest concentration in *P. gingivalis* PI groups. The decrease of IL-10 concentration is an important factor in the control of local inflammatory states associated with premature labor and CA because the reduction of this cytokine during the final stages of pregnancy with the presence of a pro‐ inflammatory state can induce PTB (Hanna et al., 2006; Azizieh and Raghupathy, 2017). However, other authors have established that the overregulation of this cytokine in the presence of infection, as another mechanism, can induce the suppression of NK and LT, thus determining the outcome of pregnancy (Arenas-Hernandez et al., 2015).

After analyzing the profiles of the different cell populations, the response identified in this study supports a macrophage activation pattern promoting an inflammatory activation with a failed regulatory mechanism which could explain the adverse outcomes identified in it. Lipopolysaccharides of Gram-negative bacteria induce the expression of MCP-1, wherein high levels are important in the development of PRM, PTB, and LBW with the presence of PI (Esplin et al., 2005). Different studies suggested that IFN-γ is primordial in the activation of macrophages by the induction of MCP-1 during labor and in the presence of APO in pregnancy (Jacobsson et al., 2003; Esplin et al., 2005). Studies in animal placenta have established that *P. gingivalis* infection also increases macrophages in placental tissue with increased local expression of mediators such as TNF-α and COX-2 (Arce et al., 2012).

The role of macrophages in childbirth is well documented; Their participation occurs in multiple ways that include: Participation in the remodeling of the cervix at delivery due to MMP secretion (such as MMP9) both in PPT and at term birth, related to an increase in macrophages residing in the decidua at the moment of delivery (Gomez-Lopez et al., 2014); It could be supposed that the presence of microorganisms (e.g., *P. gingivalis*) is related to a dysregulation of the system by activation of the previously described proinflammatory mechanisms, which could trigger labor prematurely.

Furthermore, the lipopolysaccharides of *P. gingivalis* induced a significant increase in the expression of COX-2, IL-8, and TNF-α in the trophoblast HTR-8 through the TLR-2/TLR-4-NF-κB signaling pathway, supporting the potential of *P. gingivalis* to induce proinflammatory effects in the placenta (Arce et al., 2012; Ao et al., 2015). The increase in MCP-1, TNF-α, and IL-21 and the decrease of IFN-γ for the *P. gingivalis* PI and with APO group suggest that *P. gingivalis* would directly activate the production of MCP-1 without having to be induced by high concentrations of IFN-γ. Likewise, IL-21 as an independent worker of Th-17 profile might be directly induced by bacteria lipopolysaccharides to modulate the activity of macrophages (Li et al., 2013). On the other hand, enhanced innate cell responses to *P. gingivalis* lipopolysaccharides with a decreased peripheral NK cell function in patients showing a relative NK cell energy have been described, which may be implicated in the pathogenesis of systemic illnesses that are linked to periodontitis (Gaudilliere et al., 2019).

Another immunological mechanism that has been proposed to explain preterm delivery corresponds to detectable levels of cytokines associated with the Th-17 profile that has been reported to be related to the degradation of the decidua and that increase in cases of PTB with CA (Saito et al., 2010). However, in the present study, these cytokines showed a reduction in the *P. gingivalis* PI with adverse event group (group 2), which could indicate that the cytokines of the Th-17 profile do not seem to be increased or related to the adverse event with PI associated with *P. gingivalis*. In contrast, it was observed that IL-17F regulating IL-17A in this same group presented a significant reduction, which may indicate that the problem is not the overproduction of proinflammatory cytokines but an alteration in the regulation of this cytokine profile.

## Conclusion

Periodontitis could be associated with clinical signs of placental infection and APO. Periodontal infection by *P. gingivalis* can induce atopobiosis (translocation) to the placenta and trigger inflammation, although a direct relationship with the occurrence of APO cannot be proven.

There is a pro-inflammatory pattern associated with labor, characterized by Th-1 profile and the activity of cytotoxic cells, which is enhanced by placental infection with UGM. However, PI associated with *P. gingivalis* suggests a switch where the balance of Th-1 profile favors an inflammatory response mediated by MCP-1 and macrophage activity as a mechanistic explanation of its possible relationship with adverse outcomes during pregnancy.

### Strengths and Limitations

One of the major limitations of this study was the size of the groups. Despite the number of patients included in the initial phase, the prevalence of PI was low and low concentrations of bacterial DNA were obtained. Although 12 species of microorganisms associated with PI were evaluated in this study, more could be accomplished using nested PCR-species-specific, sequencing and metagenomic techniques which could provide more information on the placental microbiome; the use of these techniques is suggested for future studies that evaluate immune response in the placenta in the presence of infection. Although the size of the groups was small, the results of this study are the first to be published on a differential pattern associated with human placental infection by *P. gingivalis* associated with APO or CA.

## Data Availability Statement

The raw data supporting the conclusions of this article will be made available by the authors, without undue reservation.

## Ethics Statement

The studies involving human participants were reviewed and approved by Institutional Ethics Committee of Universidad El Bosque (PCI-2014-18). The patients/participants provided their written informed consent to participate in this study.

## Author Contributions

The authors certify that they have participated sufficiently in the work to take public responsibility for the appropriateness of the design and method of review, as well as, the reviewing of the manuscript and approve it for publication. All authors contributed to the article and approved the submitted version.

### Conflict of Interest

The authors declare that the research was conducted in the absence of any commercial or financial relationships that could be construed as a potential conflict of interest.
