# Supplementary material for: Porphyromonas gingivalis Placental Atopobiosis and Inflammatory Responses in Women With Adverse Pregnancy Outcomes
Source: Front Microbiol. 2020 Dec 2;11:591626. doi: 10.3389/fmicb.2020.591626 (PMC7738622; doi:10.3389/fmicb.2020.591626)
Supplement: Supplementary file 1 [file Table_1.pdf]

Supplementary Table 1. Periodontal status and distribution of microorganisms in subgingival dental plaque according to the study groups.

	Control<br>*n*=17	*Pg* + APO+<br>*n*=5	*Pg* + APO-<br>*n*=4	UGM + APO +<br>*n*=5	PI - APO +<br>*n*=9
**Plaque index**	0.35 (0.19-0.5)	0.56 (0.49-0.89)	0.85 (0.73.-0.92)	0.42 (0.25-0.58)	0.30 (0.23-0.37)
**Gingival index**	0.27 (0.12-0.38)c	0.49 (0.44-0.56)	0.85 (0.73.-0.92)[a,d,e]	0.27 (0.25-0.38)[c]	0.24 (0.11-0.36)[c]
**Bleed on probing**	0.26 (0.12-0.38)	0.37 (0.30-0.56)	0.87 (0.73.-0.94)[a,d,e]	0.18 (0.16.-0.56)[c]	0.24 (0.11-0.37)[c]
**Clinical attachment level**	0.12 (0.05-0.33)	0.25 (0.09-0.40)	0.61 (0.41.-1.04)	0.07 (0.06.-1.02)	0.18 (0.03-0.41)
**Pocket depth**	2.37 (2.28.-2.56)	2.28 (2.21-2.48)	2.64 (2.55.-2.70)	2.38 (2.25.-3.13)	2.31 (2.27-2.31)
***P. gingivalis* n (%)**					
Presence **	0 (0,0%)	3 (60,0%)	3 (75,0%)	2 (40,0%)	0 (0,0%)
***T. forsythia* n (%)**					
Presence**	0 (0,0%)	0 (0,0%)	0 (0,0%)	2 (40,0%)	0 (0,0%)
***T. denticola* n (%)**					
Presence**	0 (0,0%)	1 (20,0%)	2 (50,0%)	3 (60,0%)	0 (0,0%)
***E. nodatum* n (%)**					
Presence**	0 (0,0%)	1 (20,0%)	2 (50,0%)	1 (20,0%)	0 (0,0%)
***A. actinomycetemcomitans* n (%)**					
Absence	17 (100,0%)	5 (100,0%)	4 (100,0%)	5 (100,0%)	9 (100,0%)
***F. nucleatum* n (%)**					
Absence	17 (100,0%)	5 (100,0%)	4 (100,0%	5 (100,0%)	9 (100,0%)

Periodontal indices are expressed in median and interquartile ranks. ***$p \leq 0.05$ by Chi^{2}test;* a) Significant differences Vs control b) Vs Pg +APO+, c) Vs Pg + APO-, d) Vs UGM + APO+, e) PI – APO +, by Kruskal–Wallis and /or U de Mann-Whitney tests.

Pg + APO+ = *P. gingivalis* (+) adverse pregnancy outcome (+); *Pg* + APO– = *P. gingivalis* (+) adverse pregnancy outcome –; UGM +APO+= urogenital microorganisms(+)adverse pregnancy outcome +; PI – APO+ = Placental infection (–) adverse pregnancy outcome (+). Control = adverse pregnancy outcome (–) without Intra-amniotic infection.
