# Supplementary material for: Porphyromonas gingivalis Placental Atopobiosis and Inflammatory Responses in Women With Adverse Pregnancy Outcomes
Source: Front Microbiol. 2020 Dec 2;11:591626. doi: 10.3389/fmicb.2020.591626 (PMC7738622; doi:10.3389/fmicb.2020.591626)
Supplement: Supplementary file 2 [file Table_2.pdf]

Supplementary Table 2. Distribution of adverse outcomes and clinical signs of placental infection in pregnant women according to the study groups.

		Control<br>*n*=17	*Pg* +APO+<br>*n*=5	*Pg* + APO-<br>*n*=4	UGM+APO+<br>*n*=5	PI – APO+<br>*n*=9
**PPROM (F %)**						
	**No**	17 (100)	4 (80)	4 (100)	3 (60)	6 (66.)
	**Yes**	0 (0)	1 (20)	0 (0)	2 (40)	3 (33.3)**
**LBW (F %)**						
	**No**	17 (100)	3 (60)	4 (100)	1 (20)	6 (66.7)
	**Yes**	0 (0)	2 (40)	0 (0)	4 (80)**	3 (33.3)
**PTB (F %)**						
	**No**	17 (100)	3 (60)	4 (100)	1 (20)	6 (66.7)
	**Yes**	0 (0)	2 (40)	0 (0)	4 (80)**	3 (33.3)
**LBW-PPB (F %)**						
	**No**	17 (100)	4 (80)	4 (100)	2 (40)	7 (77.8)
	**Yes**	0 (0)	1 (20)	0 (0)	3 (60)**	2 (22.2)

***p < 0.05 statistically* significant difference. Clinical signs of infection included uterine hypersensitivity, fever, maternal tachycardia, fetal tachycardia and fetid flow.

Pg + APO+ = *P. gingivalis* (+) adverse pregnancy outcome (+); *Pg* + APO– = *P. gingivalis* (+) adverse pregnancy outcome –; UGM +APO+= urogenital microorganisms(+)adverse pregnancy outcome +; PI – APO+ = Placental infection (–) adverse pregnancy outcome (+). Control = adverse pregnancy outcome (–) without Intra-amniotic infection.
